# Pleasure and Iron

**Published:** 1876-01

**Authors:** 


### PLEASURE AND IRON.

"Iron will do as a medicine, and potash is all very well, but a small bit of pleasure brings more profit."

Many a doctor has been cheated out of a nice long bill, and then has profoundly called attention to the almost miraculous effects that his iron and potash have had, when, about this season of the year, he has found patients slipping away from him, throwing off their diseases and taking a new lease of life. Have then, the turkeys, ducks and geese, mince-pie, rich pudding, confectionery and over-loaded stomachs, which the season brings, such magical restorative properties? Hardly! The doctors thrive—in more ways than one—on such things as these. When thoughts of them come, with half-closed eyes, they smile in anticipation, while they gently rub their open hands over their capacious epigastrium, and who shall say it is an accident, that on its rounds it lingers affectionately while passing over the pocket devoted to the reception of loose change. Certainly, it is not the food that is to draw invalids to new life. But this is the season of merry-making and good-cheer, when every one forgets himself—if he ever does —and takes pleasure in doing for others. Now many persons are sick because they are constantly thinking of themselves, expecting and searching after pains, and to such, this season, when they have no time to brood over their fancied ills, comes like a godsend, for they forget to be sick, endeavoring to make others happy. From inaction, they had become depressed in spirits, listless and melancholy, dejected and despondent, and as a consequence ill-conditioned in body. For such, iron and potash are all very well, but a good stirring-up is better, and if they do nothing for others, some one comes to them with a glad surprise or some token of regard that arouses them from their moping lethargy. A pleasant surprise now and then is often a better stimulant than any found in the pharmacopia. Anything, whether doing for others or being done for, that makes invalids—and especially those imagination-sick—forget themselves, gives them a powerful impulse toward good health.

From Christmas day to the second of January, is surely the merry time of the year, and in this week of festivities—the beauty and the virtue of which is that we keep it for somebody else than ourselves—many a self-sick one has forgotten self ailments and found new joy in living and doing. While the doctor is boasting another victory for physic, his bottles and powders have been thrown out of the window, and he left sighing, "Othello's occupation's gone."

